# Breast Milk and Solid Food Shaping Intestinal Immunity

**DOI:** 10.3389/fimmu.2015.00415

**Published:** 2015-08-19

**Authors:** Sara M. Parigi, Maria Eldh, Pia Larssen, Susanne Gabrielsson, Eduardo J. Villablanca

**Affiliations:** ^1^Translational Immunology Unit, Department of Medicine Solna, Karolinska Institutet and University Hospital, Stockholm, Sweden

**Keywords:** oral tolerance, breast milk, dietary compounds, microbiota, intestinal immunity

## Abstract

After birth, the intestinal immune system enters a critical developmental stage, in which tolerogenic and pro-inflammatory cells emerge to contribute to the overall health of the host. The neonatal health is continuously challenged by microbial colonization and food intake, first in the form of breast milk or formula and later in the form of solid food. The microbiota and dietary compounds shape the newborn immune system, which acquires the ability to induce tolerance against innocuous antigens or induce pro-inflammatory immune responses against pathogens. Disruption of these homeostatic mechanisms might lead to undesired immune reactions, such as food allergies and inflammatory bowel disease. Hence, a proper education and maturation of the intestinal immune system is likely important to maintain life-long intestinal homeostasis. In this review, the most recent literature regarding the effects of dietary compounds in the development of the intestinal immune system are discussed.

## Introduction

The gastrointestinal system is one of the largest vulnerable surfaces of our body. It is continuously facing the external environment, including microbiota, nutrients, metabolites, pollutants, and harmful pathogens. To maintain intestinal homeostasis, the immune system is able to induce tolerance to innocuous food antigens while it may also recognize pathogenic bacteria to mount an inflammatory immune response. The education and maturation of the intestinal immune system is the result of millions of years of co-evolution with host-specific microbiota and dietary intake. This results in mutual benefits represented by co-habitation and at the same time it provides protection against pathogens. Disruption of these homeostatic mechanisms can result in undesired immune reactions leading to intestinal disorders, such as inflammatory bowel disease (IBD), including ulcerative colitis (UC) and Crohn’s disease (CD). At birth, the transition between the sterile environment of the uterus and the external microenvironment exposes our body to colonization with maternal microbiota and food antigens, which are initially delivered through breast milk. Hence, the newborn’s immunity relies upon antibodies and other breast milk components (discussed below) passed on from their mothers. At weaning and upon introduction of solid food in the diet, the dynamic equilibrium that allows the homeostatic co-habitation with non-self antigens is continuously maintained through complex mechanisms, involving the constant shaping and education of the immune system. Food nutrients and bacterial metabolites may have a direct effect on the maturation/differentiation of immune cells. In turn, the immune system exerts active oral tolerance mechanisms to prevent reactions against food antigens and to maintain a tolerogenic environment.

This review provides insights to how the intestinal immune system is educated by oral intake of antigens before and after weaning. We will focus our attention on the impact of breast milk and diet during the development of the intestinal immune system.

## The Intestinal Immune System: Overview

**Figure 1:**
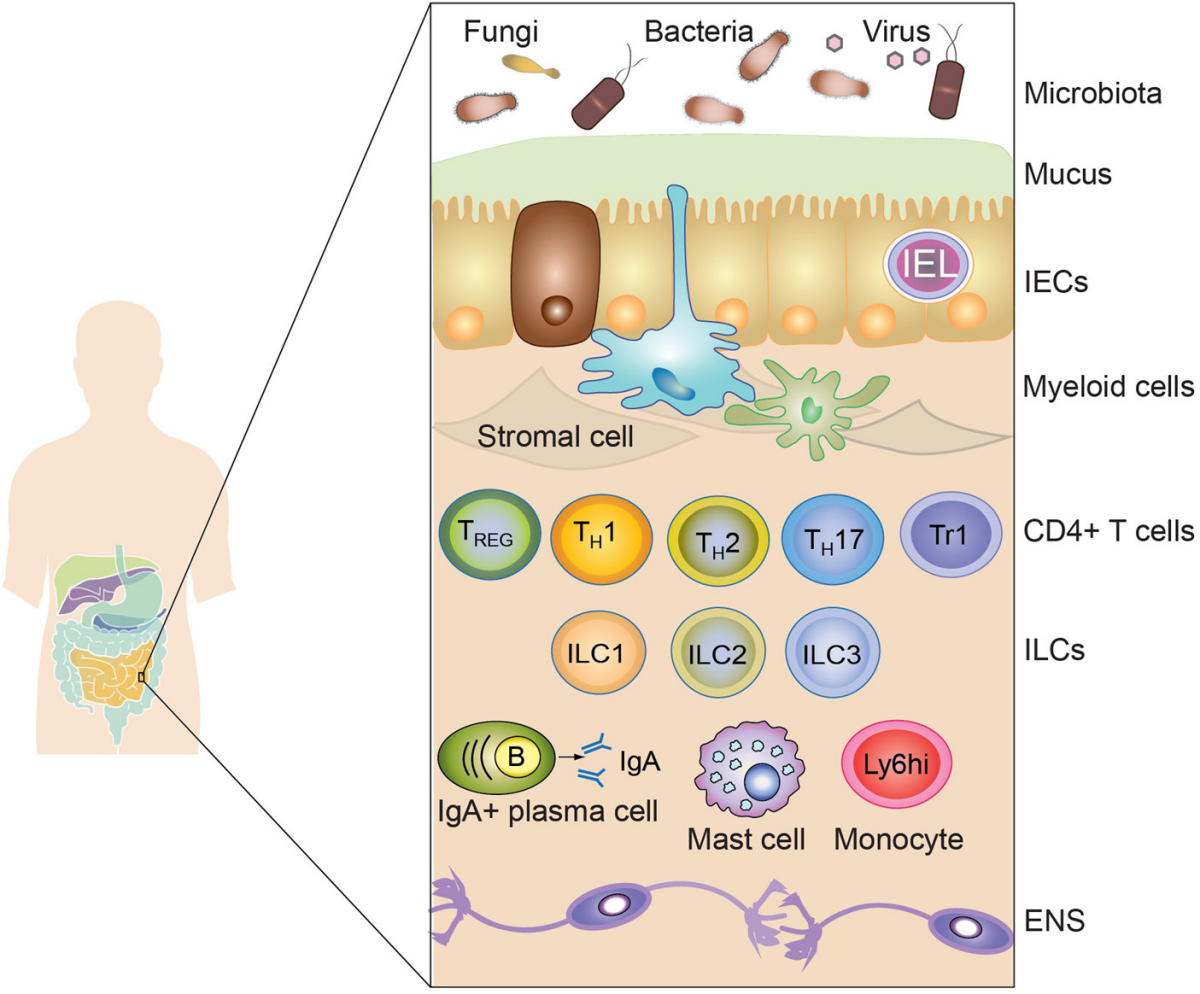
**The intestinal immune system**. The intestinal immune system is physically separated from the microbiota and dietary compounds by a single layer of intestinal epithelial cells (IECs). Intraepithelial lymphocytes (IEL), residing in the paracellular space between epithelial cells, contribute to the maintenance of the mucosal barrier and to the protection against pathogens. The lamina propria is connective tissue constituted by stromal cells, blood vessels, nerves, and immune cells. Macrophages (Myeloid cells depicted in blue) and dendritic cells (Myeloid cells depicted in green) are strategically located adjacent to the epithelial layer, sampling luminal antigens and orchestrating the innate and adaptive immune response. Other innate immune cells are also present in the lamina propria, including mast cells, monocytes, neutrophils, and eosinophils (not shown). T and B cells (mainly IgA-producing plasma cells) also accumulate in the lamina propria after being primed in the draining lymphoid tissues. Different subsets of CD4^+^ T cells are found in the lamina propria, such as regulatory T cells (Foxp3-expressing T_REG_ and Tr1) and effector cells (Th1, Th2, and Th17). Finally, innate lymphoid cells (ILC), divided in three main subsets (ILC1, ILC2, and ILC3), are vastly enriched in the gastrointestinal mucosa participating in the protection against pathogens and in the maintenance of intestinal homeostasis. Surrounding the lamina propria and the muscularis mucosa (not shown), the submucosa and the muscularis externa contain nerves belonging to the enteric nervous system (ENS).

The intestine is a complex tissue involving a single layer of intestinal epithelial cells (IECs) separating the external environment from the mammalian host. At the luminal side of the IECs layer, a high concentration of potential antigens, in the form of dietary compounds and commensal microbiota, is found, influencing mammalian physiology. At the opposite side, the intestinal lamina propria contains a diverse array of evolutionary ancient immune cells, including mononuclear phagocytic cells and lymphocytes, the associated enteric nervous system and stromal cells (Figure 1). The single layer of epithelial cells that separates the intestinal lumen from the lamina propria contains large numbers of lymphocytes with high antimicrobial and cytotoxic capacity. All these different players interact with each other in order to maintain the proper function of the digestive system, whereas failure in keeping homeostasis has been associated with the etiology of intestinal disorders, such as IBD. In the following section, we will introduce the main immune populations present in the intestinal lamina propria and their role in maintaining homeostasis. Then, we will discuss the development of the immune system shaped by breast milk and digested food.

### Intestinal epithelial cells

The main function of the IEC layer is to provide a physical and biochemical barrier to the external environment. The maintenance of intestinal homeostasis by IECs has been extensively reviewed elsewhere in Ref. (1). In brief, the IECs are composed of Paneth cells, goblet cells, enteroendocrine cells, and enterocytes, which collectively contribute to create the first line of defense against microbial invasion. This consists of antimicrobial peptides (AMPs) and the mucus layer. Microfold cells (M cells) are epithelial cells specialized in the sampling and presentation of luminal antigens to the mucosal immune system. M cells are strategically overlaying intestinal lymphoid structures, such as Peyer’s patches (PPs) and isolated lymphoid follicles (ILFs). Finally, stromal cells and stem cells, which continuously renew the intestinal epithelium, reside together with Paneth cells within tubular invaginations of the intestinal epithelium, called crypts.

### Mononuclear phagocytes

Mononuclear phagocytes including macrophages (Mφ) and dendritic cells (DCs) are among the most abundant cell types within the intestine. They are strategically located throughout the lamina propria just underlying the single layer of intestinal epithelial cells. Mononuclear phagocytes are sampling luminal content, orchestrating both innate and adaptive immune responses. Under steady state conditions, the lamina propria contains two developmentally different CD11c-expressing primary subsets of mononuclear phagocytes based on the reciprocal expression of the integrin αE (CD103) and CX_3_CR1. CD11c^hi^CD103^neg^CX_3_CR1^+^ (CX_3_CR1^+^) cells are considered to be Mφ due to their stationary nature and low stimulatory abilities (2). On the other hand, CD11c^hi^CD103^+^CX_3_CR1^neg^ (CD103^+^) cells are considered to be *bona fide* DCs due to their abilities to migrate to the draining lymph nodes and initiate effective immune responses [reviewed in Ref. (3)]. In addition, these subsets of mononuclear phagocytes have different functions and they cooperate in order to maintain intestinal homeostasis. For instance, CX_3_CR1^+^ Mφ are specialized in antigen capture from the lumen, however they do not migrate to the mesenteric lymph node (MLN) in steady state conditions (4). By contrast, CD103^+^ DCs are inefficient in capturing luminal antigens, whereas they efficiently migrate out of the lamina propria to the MLN in a CCR7-dependent manner. Furthermore, CD103^+^ DCs are able to produce TGF-β and retinoic acid (RA), which equip these cells with the ability to generate inducible regulatory T cells (iT_REG_) (5, 6). These iT_REG_ are conserved between species (5–7). Induction of gut-homing T_REG_, likely by RA-producing CD103^+^ DCs, is a crucial step during the establishment of oral tolerance (discussed below) (8, 9). Together, these cells play a crucial role in distinguishing between innocuous and pathogen-derived antigens and drive both pro- and anti-inflammatory processes. For example, CD103^+^ DCs selectively express the αv integrin, which is crucial to activate latent TGF-β (10). Activation of latent TGF-β by the αv integrin is physiologically relevant as observed in mouse models lacking αv integrin in the myeloid compartment. These mice develop spontaneous colitis associated with decreased intestinal T_REG_ (11). In addition, CX_3_CR1-deficient Mφ show decreased T_REG_ expansion, commonly observed during the establishment of oral tolerance (9). CX_3_CR1-deficient mice lack dendrite transepithelial extrusions and have impaired luminal antigen sampling, which result in reduced production of IL-10, typically released upon macrophage sensing of food and/or commensal-derived antigens (9, 12).

Although IL-10 is active in multiple immune cells, including lymphocytes, myeloid cells, and intestinal epithelial cells, it seems that Mφ are the main IL-10 cell target in order to maintain intestinal homeostasis. In fact, mice lacking IL-10Rα, specifically in CX_3_CR1^+^ Mφ, develops spontaneous colitis (13). This is in agreement with the hyperproduction of inflammatory cytokines and decreased ability to induce CD4 T cells observed by Mφ derived from patients with loss-of-function mutations in IL-10R genes (14). Notably, IL-10 depletion specifically in CX_3_CR1^+^ Mφ does not result in intestinal inflammation (13), suggesting redundant and/or compensatory sources of IL-10, most likely by type 1 regulatory T cell (Tr1). Hence, these data suggest a model in which Mφ are required to sense IL-10, which might be produced by several different cell types, to become a main tolerogenic cell with a crucial role in intestinal homeostasis. The severity of disease observed in patients with impaired IL-10 signaling underscores the critical role of Mφ and IL-10 at the intestinal barrier. However, the downstream IL-10 signaling pathways involved in imprinting Mφ, with potent tolerogenic properties, are still poorly understood.

### Lymphocytes

Naïve B and T cells that accumulate in the intestinal mucosa are primed in gut-associated lymphoid tissues (GALT), such as PPs and mesenteric lymph nodes (MLN). Upon priming within GALT, activated T cells acquire the ability to home to the intestine by expressing the gut-homing chemokine receptor 9 (CCR9) and integrin α4β7. These CCRs bind to the chemokine CCL25 and to the mucosal vascular addressin cell-adhesion molecule (MAdCAM-1), respectively (15, 16), both of them expressed in the small bowel lamina propria. Once lymphocytes, including IgA-producing plasma cells and CD4^+^ T cells, enter the mucosa they mainly distribute in the lamina propria, with the exception of CD8^+^ T cells that preferentially migrate to the epithelium (17). CD4^+^ T cells are divided into subsets, the most abundant found within the intestinal lamina propria are IL-17 producing T helper cells (Th17), Th1 and Regulatory T cells (T_REG_). T_REG_ include two types of CD4^+^ T cells; forkhead box P3 (Foxp3)^+^ T cells and Tr1 cells, which provide the foundation of the tolerogenic immune response. Their relevance during the establishment of intestinal immune homeostasis has been demonstrated by mutations in human *FOXP3*, which are associated with the fatal autoimmune disorder Immunodysregulation Polyendocrinopathy Enteropathy X-linked (IPEX) syndrome (18, 19). In mice, disruption of Foxp3^+^ T_REG_ development and/or function results in intestinal-associated autoimmune and inflammatory disorders (20), likely due to the inability to suppress immune responses against commensal bacteria. Human and mouse Tr1 cells can be identified by the surface markers CD49b and LAG-3 as well as the production of high IL-10 levels (21). Importantly, transfer of Tr1 cells into colitic mice prevents intestinal inflammation, highlighting their immunosuppressive role (21). T_REG_ exert their immunosuppressive functions through several mechanisms, including the production of inhibitory cytokines, such as IL-10 (22), metabolic disruption, expansion of innate lymphoid cells (ILC)1-like NK cells and ILC2s (23, 24), and modulating DC functions (25), all of which have the final outcome of limiting expansion of antigen-specific T cells.

In contrast, Th1 and Th17 cells are mostly associated with pathogenicity during chronic inflammation. Indeed, important efforts to develop drugs targeting the IL-17-Th17 pathway to treat autoimmune diseases have been made (26). In agreement with a pathogenic role, Th17 generation require encounters with antigen presenting cells (APCs) within a pro-inflammatory microenvironment, characterized by the presence of IL-23, IL-6, and IL-1β (27). In addition, intestinal Th17 cells also promote tissue repair and protect mucosal barriers against pathogen colonization, hence contributing to maintain intestinal homeostasis (28). Some studies suggest that their protective and/or pathogenic function depends on plasticity, in which pathogenic Th17 cells might gain immunosuppressive functions (28). In a recent study, using a mouse model of Th17 fate-mapping combined with reporters to visualize the appearance of T_REG_ *in vivo*, it was demonstrated that Th17 cells can transdifferentiate to IL-10-producing T_REG_ to eventually contribute to the resolution of inflammation (29).

### Innate lymphoid cells

Innate lymphoid cells (ILCs) belong to the lymphoid lineage. In contrast to B and T cells, ILCs lack antigen receptors and do not undergo clonal selection when stimulated. This newly described cell type is vastly present at mucosal surfaces, in particular throughout the GI tract and within ILFs. ILCs have been classified into three subtypes based on their cytokine production and expression of determined transcription factors (30). Group 1 ILCs (ILC1s) are characterized by their expansion in response to IL-12, IL-15 and IL-18, and their production of type 1 cytokines, such as IFNγ and the transcription factor T-bet. Although natural killer (NK) cells also express IFNγ and T-bet, ILC1s are different in that they lack cytolytic activity and possess a separate developmental pathway compared to NK cells. Furthermore, fate mapping of ID2^+^ or PLZF^+^ precursor cells distinguished ILC1 from NK cells (31–33). Group 2 ILCs (ILC2s) require the GATA-binding protein 3 (GATA3) and ROR-α transcription factors, expand in response to IL-25, IL-33, and TSLP and produce IL-5, IL-9, and IL-13, typically associated with Th2-responses. Group 3 ILCs (ILC3s) depend on the RA receptor-related orphan receptor-γ (RORγt) transcription factor, respond to IL-1β and IL-23 produced by myeloid cells and secrete high amounts of IL-17A and IL-22, as well as GM-CSF and lymphotoxins (34). Thus, due to their similarities, ILCs are considered the innate counterpart of T helper cells.

Innate lymphoid cells are important mediators of inflammation and exert protection against pathogens, but they are also key drivers of homeostasis under steady state conditions. For instance, ILCs have been associated with worsened *Helicobacter hepaticus*-triggered intestinal inflammation (35). In accordance with this model of pro-inflammatory activities, depletion of ILCs results in significant amelioration of colitis in the *H. hepaticus*-triggered intestinal inflammation (36) and in the *Tbx21*^−/−^*Rag2*^−/−^ ulcerative colitis (TRUC) model (37). Despite the implications with disease, ILC3s have also been implicated in critical protective roles, including defense against the pathogenic bacteria *Citrobacter rodentium* (38, 39), containment of lymphoid-resident commensal bacteria (40) and induction of mucin and antimicrobial peptides (e.g., RegIIIβ and RegIIIγ) (41, 42). Most of these protective functions rely on the production of IL-22 upon stimulation with either IL-23 or IL-1β. ILC3s produce large amounts of this cytokine and administration of exogenous IL-22 in ILC-depleted mice is sufficient to restore homeostasis. In addition, ILC2s and ILC3s express major histocompatibility complex class II (MHCII) (43), hence being able to present antigens to CD4^+^ T cells. In the intestine, the absence of MHC-II on ILC3s results in expansion of commensal-specific CD4^+^ T cells, which eventually leads to intestinal inflammation (44, 45). MHC-II expressing ILC2s induce the production of IL-2 and IL-4 by CD4^+^ T cells, thus potentiating type 2 immune responses (46). Furthermore, it has recently been proposed that the few resident memory T cells with pathogenic features within the non-inflamed intestine might function as antigen-specific sensors, whereas ILCs might serve to amplify T-cell mediated antigen-specific responses (47). Thus, these newly described cell types play critical roles in maintaining intestinal homeostasis through, among others, shaping the intestinal flora.

At early stages in life, the intestinal immune system is underdeveloped and undergoes immune maturation upon contact with dietary compounds and the microbiota. In agreement with this, germ-free and antibiotic-treated mice show reduced maturation of the intestinal immune system, as seen by decreased numbers of intestinal Th17 cells (48, 49), impaired production of antimicrobial peptides, and reduced IgA secretion, all deficiencies that are rescued upon bacterial colonization (50). Besides regulating the diversity of the microbiota, dietary compounds may directly influence the development of the immune system. In the following section, we discuss how the external environment, including dietary compounds and the commensal microbiota, shapes the intestinal immune system.

## Breast Milk Shaping Mucosal Immunity

**Figure 2:**
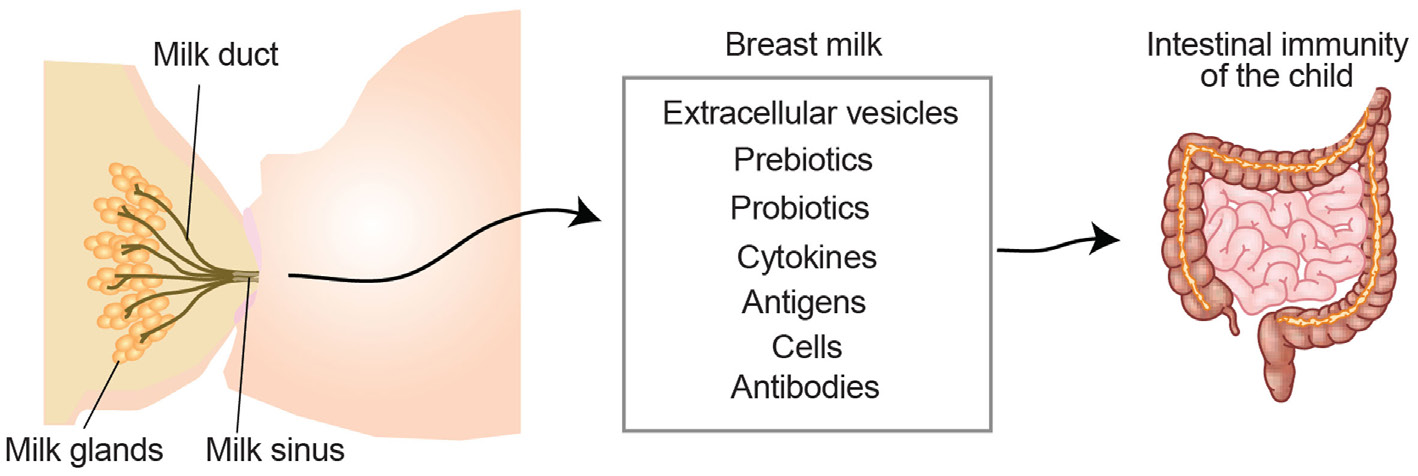
**Breast milk-derived compounds with the potential to contribute to the development of the child intestinal immune system**. Breast milk contains extracellular vesicles such as milk fat globules and exosomes, which can present antigens in a tolerogenic setting. Pre- and pro-biotics influence the microbiota contributing to symbiosis and intestinal protection. Milk-derived cytokines, which may differ depending on the health status of the mother, have the potential to directly promote the immune system development of the child. Milk-derived antigens have been shown to both induce tolerance and allergies in the lactating child. White blood cells from the mother can also be transferred through the milk and exert functions in the child (e.g., production of cytokines or antibodies). Milk-derived antibodies are originated from the mother and support the child with a passive immunity during the first months of life.

Breast milk is not only a primary source of nutrition, it also helps the child develop a proper immune system. Breast milk is a complex body fluid composed of a large diversity of molecules, cells, and extracellular vesicles. Already in 1892, Paul Ehrlich showed that immunity against plant toxins was transferred to the fetus *in utero* and via breast milk. This turned out to be antibodies protecting the child, leading to the term “passive immunity,” which gave him the Nobel Prize and paved the way for modern immunology. In addition to protection to pathogens, breast milk also contributes to establish intestinal tolerance, likely through the action of breast milk-derived components (Figure 2), such as immune cells, cytokines, anti-bacterial proteins, probiotics, and extracellular vesicles (51, 52). The role of breast milk in shaping intestinal immune maturation and contributing to immunological tolerance will be discussed below.

### Duration of breast-feeding

There is great controversy, especially in industrialized countries, regarding the duration of breast-feeding and whether partial or exclusive breast-feeding would be more beneficial. The World Health Organization (WHO) and UNICEF recommend exclusive breast-feeding up to 6 months of age [WHO, Guiding Principles for Feeding Non-Breast-Fed Children Aged 6–24 Months of Age WHO, Geneva (2005)], and countries of the European Union and EFTA recommend exclusive breast-feeding for at least the first 4–6 months (53). In wealthy countries, where early infections, including life-threatening diarrhea are scarce, exclusive versus partial breast-feeding is likely not as important as in developing countries. In contrast, some believe that food allergies can be reduced by introducing solid food under the protection of breast milk.

Several studies have addressed the question regarding the correlation between breast-feeding and allergy development. Most of these studies suggest that breast-feeding may protect against allergies, at least when comparing with formula-feeding (54, 55). However, differences in genetics, exposure to allergens, pathogens, commensals, and smoking, as well as lifestyle, may lead to different conclusions. For example, in a Danish study exclusive breast-feeding duration was associated with increased risk of eczema development in children under the age of 2. In contrast, the risk for developing wheezy disorder in these children was reduced, even with maternal heredity for asthma (56). The exact immunological mechanisms are not easily extracted from epidemiological correlations. In addition, in a review revising the literature regarding breast-feeding and allergies between 1966 and 2001, only 56 out of 4323 studies were considered conclusive (57).

Studies on the relation between autoimmune diseases and breast-feeding are fewer but seem more conclusive for a protective effect of breast-feeding. Retrospective [reviewed in Ref. (58)] and prospective studies have shown that longer breast-feeding is protective for type 1 diabetes (59). Also IBD [meta analysis in Ref. (60)] and multiple sclerosis (61) prevalence are lower in individuals with longer breast-feeding history, indicative of an anti-inflammatory role of breast milk.

### Immune-regulating components in breast milk

During pregnancy, the mammary gland undergoes large modifications in order to transform into a milk-secreting organ. A branching network of ducts that are made up by a single epithelial cell layer and ending up in lobulo-alveolar cavities make up the lactating mammary gland. Surrounding the alveoli are myoepithelial cells, which when stimulated by oxytocin release, contract and enable milk ejection into the cavities (62). The first milk, from delivery to 5 days post-partum, is called colostrum and is produced in reduced amounts and the composition is lower in fat and lactose compared to mature milk (63–65). This is compensated by the colostrum being rich in immunologic components such as lactoferrin, secretory IgA (sIgA), developmental factors (e.g., EGF, TGFβ1, TGFβ2), and other cytokines (65, 66). Hence, the primary function of colostrum might not be nutritional but rather immunologic and trophic (64, 65). Some of the components with potential immunoregulatory functions are discussed below.

#### Antibodies

The majority of antibodies in milk are IgA, but breast milk also contains IgG and IgE. IgA serves as a first line of defense of mucosal sites to provide a passive immunity to the child who has no IgA production of its own during the first months after birth (67–70). Interestingly, specific antirotaviral IgA has been found in colostrum and mature breast milk and furthermore in infant stool. These antirotaviral antibodies are likely to provide a passive protection in the child (71). Therefore, the transferred sIgA is an important barrier protecting the newborn from intestinal and respiratory pathogens (72). Other studies have shown that maternal sIgA affects the development on gut microbiota in the offspring (73), which in turn affects the immune development. In relation to allergy, it was shown that high IgA levels in the feces of the infant were associated with lower risk for allergy development (74). In addition, allergen-specific immunoglobulins have been detected in neonates after allergen exposure in mice (75) and humans (76). Whether the IgA work as a decoy receptor for the allergen, or if it is the effect on the microbiota, is still unresolved.

Mouse models support the role for breast milk immunoglobulins and/or B cells in the development of the immune system and allergy development. In a B cell deficient mouse model, it was shown that maternal B cell immunity is important for protecting the offspring against allergic airway disease (77). The transfer of immunity was shown to be accompanied by transfer of antigen-specific IgA and IgG. Another study demonstrated that recombination activating gene-2 (Rag-2) deficient mouse pups (lacking B and T cells) had different immune responses if they received milk from wild type or Rag-2^−/−^ mothers (78), indicating that the lymphocytes from the mother play a crucial role transferring immunity to the progeny. Whether the effects were due to immunoglobulins, cells or both could not be distinguished in this study and remains to be investigated (78).

#### Cells

Cell exchange between the mother and the fetus occurs already *in utero*, and this results in microchimerism in the child (79). This also continues after birth through breast milk (80). The most abundant cell type in human breast milk is leukocytes, including granulocytes, monocytes, lymphocytes, and Mφ (81, 82). In colostrum, Mφ are the major cells (40–50%) followed by neutrophils (40–50%) and lymphocytes (5–10%) (83–85). Interestingly, breast milk can also contain pluripotent stem cells (86), although their fate within the newborn needs to be investigated. Several studies, in mice, rats and primates have shown that milk-derived leukocytes might be taken up by the offspring where they can survive and exert their functions in the newborn stomachs. The less acidic newborn stomachs might provide a more permissive microenvironment for leukocyte survival compared to adult stomachs (87–89).

Breast milk-derived Mφ are more comparable to tissue resident Mφ, as they have higher HLA-DR expression and are able to carry MHC class II antigens to a higher extent than the monocytes found in blood (90). In addition, they express activation markers such as Leu-M3 and Leu-M5 (90). Neutrophils also seem to be more activated in milk compared to those in blood (91). Moreover, breast milk-derived Mφ also carry intracellular IgA, which can be released upon stimulation (92). Breast milk-derived T cells show a more terminally differentiated and memory phenotype compared to circulating T cells (93). Most breast milk-derived T cells express mucosal homing markers, such as the integrin αE (CD103), integrin β7, CD49d and CCR9 suggesting that they were primed in GALTs and then migrated to the mammary gland (94, 95). Furthermore, γδ^+^ T cells, which are commonly found in the intestinal mucosa, are also enriched in breast milk compared to blood (96). Breast milk-derived B cells are also different to those circulating in blood. For instance, breast milk-derived B cells show a more activated phenotype compared to those found in peripheral blood (97, 98). Interestingly, IgA-secreting B cells localized in the intestine can home to the mammary glands (99). This phenomenon seems to be dependent on CCR10 (or CCR3), since up-regulation of CCL28 in the mammary gland leads to the recruitment of antibody-secreting cells and to an increased production of IgA in the milk that is blocked using anti-CCL28 antibodies (100). This mechanism might provide breast milk with intestinal microbiota-specific IgA, but other soluble factors from the B cells such as cytokines could also play a role. Thus, these observations suggest that lymphocytes might be educated at intestinal sites to recognize commensal bacteria, traffick to the mammary gland and transfer commensal bacteria-specific immune responses through breast milk to the newborns.

#### Antigens (Food Allergens/Respiratory Allergens)

Food allergens have been extensively detected in human breast milk. A randomized double-blind cross-over study showed that ovalbumin (OVA) can be detected in human breast milk in a dose-dependent way, up to 8 h after egg intake (101). Other food allergens can also be transferred via breast milk, such as cow milk proteins (102) and the peanut allergen Ara h 6, which can be rapidly transferred to breast milk just 10–20 min after ingestion of peanuts (103). This suggests that food allergens are not fully degraded in the digestive tract of the mother, and further that active allergens can be transferred via breast milk. A comparison between suckling and formula-fed rat pups showed that early introduction of food antigens together with milk shifted the cytokine milieu in a tolerance inducing way (104), which suggests an important early mechanism for development of oral tolerance. Breast-fed pups had higher IFN-γ levels and elevated CCR7^+^ and Foxp3^+^ cell numbers compared to formula-fed rats (104). This in turn led to an increased secretion of IL-10 by T_REG_ proposing that these mice have a greater potential to induce a tolerant local gut environment. In agreement, Yamamoto et al. showed in a mouse study that providing lactating dams with food antigens led to induction of oral tolerance to food antigens in the pups (105). Particularly pronounced protection was observed in the offspring of sensitized dams, who had higher antigen specific IgG1 levels in the breast milk. Subsequently, the offspring of the sensitized mothers showed higher amount of antigen specific IgG1 levels in their plasma. Transferred antigens have also been shown to induce tolerance in the offspring. This was observed in mouse pups that were given human breast milk containing peanut allergen, which resulted in partial oral tolerance to the antigen (103). Not only food antigens have been found in human breast milk, but also respiratory antigens such as Der p 1 allergen from house dust mite (106). However, in contrast to food antigens, Der p 1 in milk was shown to promote sensitization when the human milk was given in a mouse model of asthma (106). This discrepancy could be due to either the xenobiotic nature of the experiment or to the nature of this antigen, as it has enzymatic activity and can damage the epithelial barrier (107). In an OVA model, Verhasselt et al. showed elegantly that OVA areosols could be transferred via milk and induce tolerance in the pups, and that this was dependent on TGF-β (108). However, it is difficult to rule out if OVA have been digested to some degree. In addition, the presence of the antigen in the gut might contribute to induce tolerance.

#### Cytokines and Other Soluble Factors

Several chemokines and cytokines, which might have a major impact on the mucosal and lymphoid tissues in the child have been found in human breast milk (109). IL-6 and TGF-β have been identified as the most abundant cytokines present in breast milk (110). For instance, breast milk-derived IL-6 has been shown to be crucial for IgA production from milk mononuclear cells (111). With potential implications in allergy, IL-4 can be detected in both colostrum and mature breast milk, with elevated levels in colostrum from allergic mothers compared to non-allergic mothers. Similarly, IL-8 is found in higher amounts in allergic mothers compared to non-allergic mothers (112). In contrast, the immunosuppressive cytokine IL-10 has also been detected in breast milk (113). *In vitro* studies have shown that an anti-proliferative effect of milk on peripheral blood mononuclear cells (PBMC) was reduced when adding anti-IL-10 antibodies, demonstrating IL-10 activity in the milk (113). Breast milk-derived TGF-β might also play an important role inducing intestinal tolerance. In agreement, breast milk-derived TGF-β acts on T cells, which eventually confer protection to develop allergies (108). Finally, IL-1 receptor (IL-1R) antagonist has been found in human breast milk. The fact that mice with colitis are protected similarly if they receive human milk or formula supplemented with IL-1R antagonist (114), suggest that IL-1R signaling can have a major role in tolerance induction in the offspring.

The soluble protein CD14 can work as a receptor for lipopolysaccharide and can be transferred through breast milk from rat dams to the pups (115). CD14 is up-regulated after bacterial exposure and is secreted by innate immune cells such as monocytes as well as epithelial cells (116). The association between low exposure to CD14 via the breast milk and increased risk for atopy has been observed in some cohorts (117) but not in others (118), and it remains to be further studied whether breast milk-derived CD14 modulates immunity in the offspring. In addition, the presence of complement factors (119), free radicals and antioxidants, such as β-carotene (120) and α-tocopherol (121) in milk are also likely to modulate the microbiota and intestinal immunity of the child.

### Factors affecting the microbiota

It has been suggested that bacteria from the maternal gut can be transported by mononuclear cells to the mammary gland and transferred to the breast-fed infant (122, 123). Human breast milk contains a large diversity of probiotic (having a positive health effect) bacteria, such as, *Lactobacillus rhamnosus, Lactobacillus gasseri, Lactococcus lactis, Leuconostoc mesenteroides*, *and Bifidobacteria*. Therefore, breast milk is an important source of bacteria for the undeveloped gut of the infant (124–127). Breast milk-derived probiotics possess the ability to positively modify the gut microbiota of the infant (e.g., to help improving the health of the infant gut). Furthermore, in a large population-based pregnancy cohort, an association has been shown between reduced risk of atopic eczema in infants and the consumption of probiotic milk of mothers during pregnancy (128). Therefore, probiotics might reduce the risk of atopic eczema (128).

Oligosaccharides are prebiotics (carbohydrates serving as nutrient for probiotics) that are abundant in milk, which also alter the gut flora (129). High levels of oligosaccharides in breast milk correlate with higher diversity of *Bifidobacterium* species, and inhibit the adhesion of pathogenic bacteria (130), hence showing protection to infection. Moreover, Lactoferrin is another component of milk with bactericidal properties against many pathogenic bacteria (131). However, Lactoferrin can also promote the growth of *Bifidobacteria* species (132), leading to an altered gut flora. The medium-chain saturated and long-chain unsaturated fatty acids present in breast milk have also been shown to be anti-microbial (133) (see above). In addition, lysozyme will also affect the gut flora of the infant since it has both anti-bacterial and anti-viral activities (134, 135). This is probably contributing to the anti-pathogenic effect of breast milk, but the effect on the commensal flora is still unclear.

#### Extracellular Vesicles

The mammary epithelial cells secrete milk through five different secretory pathways (62), whereof the milk fat globule pathway is specific for mammary epithelium (136). This pathway is a budding process, which generates lipid-containing vesicles, milk fat globules (MFG) surrounded by a lipid membrane, and is a way to release the fat without the risk of clumping. The MFGs has a protective effect against microorganisms (137–139), which comes partly from the triacylglycerol rich-core, but also from the membrane, which contain pH resistant glycoproteins such as mucin-1 (MUC-1), MUC-X, and lactadherin (138, 139). MUC-1 has been shown to attenuate epithelial inflammation (140), in agreement with the role of mucins creating a protective shield in the newborn gut against bacteria and viruses (141). Another type of extracellular vesicle that can be found in breast milk is exosomes (142), which are nano-sized membrane vesicles, which originate from the endosomal pathway. Exosomes contain a large diversity of lipids, proteins, and several kinds of RNA (143, 144). Importantly, the presence of mRNA (145) and miRNA (146) has been detected within breast milk-derived exosomes. Some of the proteins are common for all exosomes, regardless of their cellular origin, such as proteins involved in multivesicular body biogenesis or membrane transport and fusion, and tetraspanins such as CD9, CD63, and CD81 (147). Exosomes also contain cell-specific proteins, such as MHC class I and II as well as co-stimulatory molecules on their surface, when derived from APCs (148).

Virtually all cells release exosomes, which have both immunostimulatory (149) and immunosuppressive functions (150). First reported to have an immunostimulatory function, were exosomes released by B cells, which express MHC class II and are therefore equipped with the ability to induce antigen-specific CD4^+^ T cell responses *in vitro* (149). Importantly, using DC-derived exosomes harboring MHC class I, induction of CD8^+^ T cell responses was shown *in vivo* (151). The ability of exosomes to indirectly or directly stimulate T cells has been documented by several other studies (152–154).

Our studies have shown that breast milk-derived exosomes are likely to have an immunosuppressive role, as observed in PBMC cultures *in vitro*, in which the presence of breast milk-derived exosomes were able to inhibit cytokine production and induce Foxp3^+^ T_REG_ (142). Whether this effect was mediated via DC or the exosomes acting directly on the T cells, is currently under investigation. Cow’s milk has also been shown to contain extracellular vesicles carrying TGF-β (155), and bovine milk exosome-derived microRNA was shown to have immunoregulatory effects in *in vitro* macrophage experiments (156), further suggesting an immunosuppressive role for breast milk-derived exosomes. An immunosuppressive function of exosomes derived from the gut epithelium has also been shown in their ability to induce antigen-specific tolerance (150). Exosomes derived from serum of OVA fed rats have been shown to induce antigen-specific tolerance in naïve recipient animals. The origin of these exosomes is believed to be the intestinal epithelium, as exosomes isolated from *in vitro* pulsed intestinal epithelium cells showed the same characteristics as the serum-derived exosomes (150). In addition to intestinal epithelium-derived exosomes, also exosomes of other cellular origins have the ability to induce tolerance. Prado et al. demonstrated that exosomes derived from BAL fluid of antigen-specific tolerized mice could inhibit allergen-induced airway inflammation (157). This was shown by decreased levels of IgE antibodies in mice pre-treated with BAL-derived exosomes from tolerized animals, compared to those treated with control exosomes. In addition, they further showed that these mice, pre-treated with exosomes from tolerized animals, also had a reduced level of IL-10 and a significantly reduced production of IL-5 (157). Whether breast milk-derived exosomes possess similar properties *in vivo* still needs to be investigated.

Although a role for breast milk exosomes in shaping the immune system of the child is plausible, only *in vitro* studies and indirect observations are currently available to support this. In a study comparing allergic and non-allergic mothers, we have shown that allergic sensitization and lifestyle of the mother affects the phenotype of breast milk-derived exosomes. In this study, it was also shown that there is a relationship between the breast milk-derived exosome profile and the sensitization of the child, suggesting that the exosomes play a role in the cell-to-cell communication between mother and child (158). We also observed that MHC class I was low in early milk, and higher in late milk, suggesting that endogenous antigens, e.g., viruses or autoantigens, might be more exposed later after birth. By contrast, since MHC class II was high early and reduced later, allergens transferred from the mother might be more presented early after birth. One could speculate that this differential exposure has evolved to expose the child to different antigens at optimal time points during development. However, new exposure patterns in modern lifestyle might have led to an imbalance and more allergy or autoimmune development. In addition, breast milk-derived exosomes carry MUC-1 and were shown to interact directly with DCs by binding to the DC receptor DC-SIGN, and furthermore blocked HIV infection of DC by blocking the entry of the virus (159). Whether this occurs *in vivo*, with possible delivery of exosome cargo to DCs in the gut or in the circulation, remains to be investigated. However, since exosomes (“tolerosomes”) also can be produced by the gut epithelium, and have in this context been shown to be able to induce tolerance, we speculate that also milk exosomes could have this effect (150), delivering tolerogenic signals to the offspring.

Upon weaning, breast-feeding is discontinued whereas dietary intake of solid food increases, exposing our GI tract to a vast range of nutrients and exogenous antigens. Dietary intake and eating habits have a profound impact in shaping the immune system and regulating the microbial composition of the gut. However, a recent study suggested that interruption of breast-feeding together with the mode of delivery (vaginal compared with C-section) are the crucial steps in the definition of microbiota assembly, rather than the introduction of a solid-food diet (160). Nevertheless, a controlled nutritional regimen is crucial for the proper maturation of a functional immune system and to allow protection against infection and intestinal or systemic disorders (161). Furthermore, paralleling the hygiene hypothesis, a “diet hypothesis” has been proposed to explain the differential incidence of allergic and chronic inflammatory diseases among countries with a similar cleanliness in the environment but with remarkable different diets (162). Education of the intestinal immune system in relation to dietary intake can be outlined into two main processes: (1) The sensing of specific food metabolites, which affects maturation, differentiation, and activity of the intestinal immune system. (2) The active mechanisms to induce tolerance toward ingested antigens, a process referred to as “Oral Tolerance”.

## Dietary Compounds Shaping Mucosal Immunity

**Figure 3:**
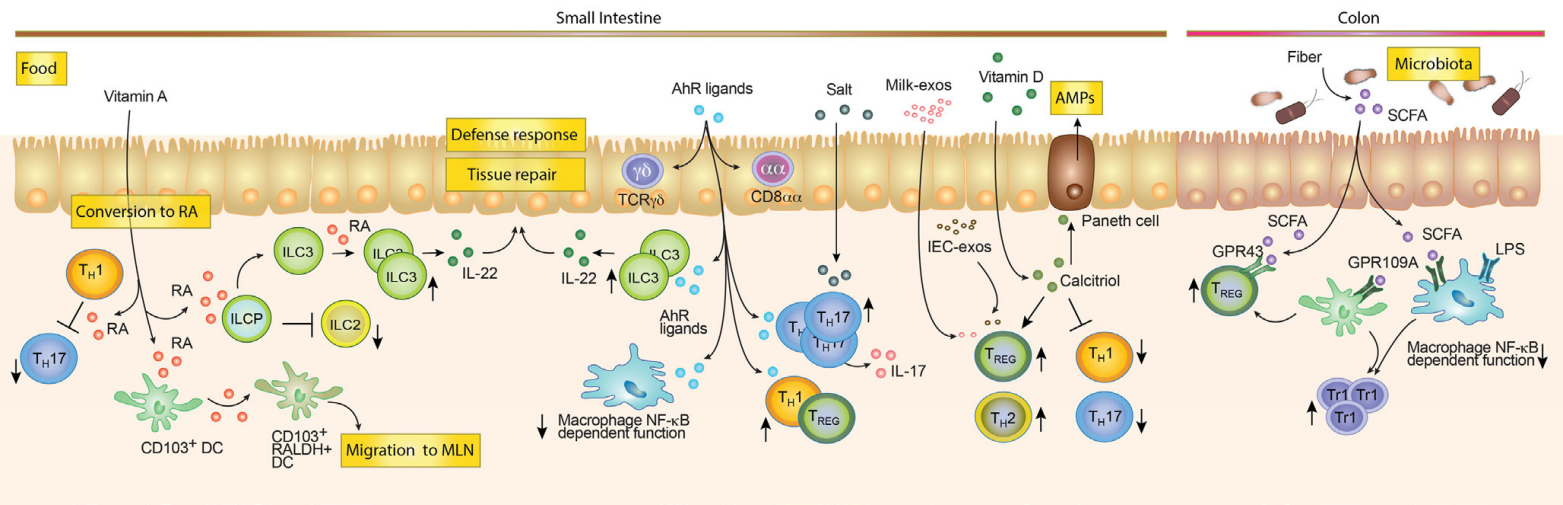
**Dietary compounds contributing to the development of the intestinal immune system**. Food components acting directly on immune cells such as Vitamin A and D, salt and AhR ligands are enriched and mostly absorbed in the small intestine. Vitamin A can be converted by RALDH expressing cells into Retinoic Acid (RA). RA can modulate the function of different cells. For example, it can inhibit Th1 cells to become pathogenic Th17 cells, block ILC2s development while promoting ILC3s differentiation and expansion and imprint DC with the ability to produce RA. While in the mesenteric lymph node (MLN), RA-producing DCs may induce gut tropism on T and B cells, as well as promoting T_REG_ differentiation (not shown). AhR ligands favor the maintenance of intraepithelial lymphocytes (e.g., TCRγδ and CD8αα T cells). Furthermore, they promote Tr1 and T_REG_ differentiation, inhibit NF-κB signaling in macrophages and promote the expansion of ILC3s. Salt rich diets and AhR ligands are associated with expansion of Th17 cells. Milk-derived exosomes (Milk-exo) and epithelial cell-derived exosomes (IEC-exo) have been shown to promote T_REG_ differentiation/expansion. Although, the precise mechanism and if this occurs in the intestine *in vivo* is still unknown. Vitamin D is converted into the active metabolite Calcitriol (1,25(OH)_2_D_3_), which may promote T_REG_ and Th2 cells while blocking Th1 and Th17 differentiation. In addition, Calcitriol enhances the production of anti-microbial peptides by Paneth cells. Dietary compounds, which require bacterial enzymatic digestion, may preferentially exert immunoregulatory functions in the colon. Dietary fibers are metabolized by the microbiota, which leads to the production of short-chain fatty acids (SCFA) metabolites. SCFA promote T_REG_ responses through GPR43 and indirectly by triggering GPR109A on colonic myeloid cells (Mφ and DCs). In addition, the anti-inflammatory effect of SCFA is mediated by the inhibition of histone deacetylases (not shown), NF-κB signaling and the inhibition of the LPS-induced inflammatory cascade.

Our body directly absorbs some dietary metabolites, while others are indigestible by the enzymatic repertoire encoded by our genome. Therefore, microbial digestion of these metabolites is needed for their absorption (163). This is likely reflected by a spatial distribution of these food metabolites along the GI tract. For instance, Vitamin A or food ligands for the AhR receptor (see below) are enriched in the proximal small intestine (e.g., duodenum). By contrast, short-chain fatty acids (SCFA), which are metabolites of indigestible fibers, require bacterial enzymes to be digested and absorbed, hence being more concentrated in the large intestine (16). Consistently the effect of these food components on the education of immune cells follows the same pattern of distribution. (16) (Figure 3). As an example, SCFA promote the generation of CD4^+^ T_REG_ cells that are enriched in the colonic tissue, whereas AhR ligands drive the differentiation of Th17 cells that in turn are more prevalent in the small intestine. These examples highlight the concept that the small intestine and colon are two different compartments composed of different metabolites, organisms and cell types. In the following sections, we will discuss how food metabolites educate our intestinal immune system. We will discuss the role of food components that are directly absorbed by our body (“Unmodified dietary components”) and dietary antigens that require bacterial metabolic processing (“Commensal-derived food metabolites”).

### Unmodified dietary compounds

Some food components do not require metabolic processing by commensal bacteria in order to be absorbed and digested by our body.

#### Vitamin A

Deficiency in Vitamin A and its metabolite retinoic acid (RA) in the diet has been associated with increased HIV pathogenesis, poor responses, and increased susceptibility to infections, both at the GI tract and in the lungs (164, 165). The dietary sources of Vitamin A encompass animal products (such as human milk, liver, and egg yolk) containing Vitamin A in the form of retinyl esters and food containing Vitamin A precursors, or carotenoids (such as yellow and green leafy vegetables). Upon absorption in the proximal small intestine, retinyl esters undergo a complex metabolic pathway that might lead to the oxidation of the Vitamin A metabolite, retinal, into RA. RA functions are then mediated by the binding to the RA nuclear receptors (RARα, RARβ, and RARγ) heterodimerized with retinoid X receptors (RXRα, RXRβ, and RXRγ) (166). The crucial role of RA orchestrating mucosal immunology began to be elucidated by the seminal article published a decade ago by Iwata and colleagues, showing that RA was necessary and sufficient to induce leukocyte trafficking to the gut mucosa (167). Subsequent work has shown that RA induces gut tropism in B cells and promotes the induction of IgA-producing plasma cells at mucosal surfaces (168). Furthermore, RA acts as an adjuvant to induce Foxp3^+^ T_REG_ and Th17 differentiation [reviewed in Ref. (169)]. In addition, recently Noelle and colleagues showed that RA action on RARα represses the reprograming of Th1 to pathogenic Th17, hence playing a role in Th1–Th17 plasticity (170). The effect of RA might depend on the microenvironment. In a pro-inflammatory milieu characterized by the presence of IL-15, RA promotes inflammatory cellular and humoral responses to fed antigens (171).

Retinoic acid might also indirectly act on the immune system. This was observed in mice reared on a Vitamin A deficient diet (VAD), which results in disruption of number and composition of the gut microbiome (172). In particular, decreased segmented filamentous bacteria (SFB) were accompanied by a reduced proportion of Th1, Th17, and Th2 cells (173).

Recently, a critical role of RA in ILC homeostasis has been demonstrated. For instance, mice reared on a VAD are characterized by increased numbers of ILC2s and reduced numbers of ILC3s as well as ILC3-derived cytokines, such as IL-17 and IL-22 (173). Interestingly, the transfer of RARα-deficient ILCs progenitors recapitulated the same phenotype, suggesting that RA intrinsically suppresses ILC2 development. Besides its role in differentiation, RA modulates IL-22 production possibly by direct engagement of the RARγ with the IL-22 promoter (174). Another recent report demonstrated that RA regulates the differentiation of lymphoid tissue inducer (LTi) cells, which are essential for the development of secondary lymphoid organs (175). As a consequence, the availability of RA *in utero* has an impact on the education of the newborn immune system and protects against infection in adulthood (175). Thus, RA plays a crucial role in ILC homeostasis, which is then reflected by increased susceptibility to bacterial infections, intestinal inflammation, and the overall fitness of the intestinal immune response (173, 174).

In adults, the sources of RA have been extensively studied and reviewed elsewhere in Ref. (176). The limiting enzymes in RA metabolism are retinal dehydrogenases (RALDHs), which are highly expressed by intestine-resident CD103^+^ DCs, epithelial cells and stromal cells, as well as by CD103^−^ CD11b^+^ DCs in the skin and lung (166). Expression of RALDH enzymes is crucial to metabolize RA, since cells lacking these enzymes are not able to produce RA (177). Interestingly, gut-homing expressing DC precursors migrate from the bone marrow to the small bowel, where they acquire tolerogenic properties (178, 179). Once DC precursors reach the intestine, they might differentiate and sense RA to induce the expression of RALDH enzymes, with the subsequent *de novo* production of RA (179). The role of RA in educating intestinal DC is in agreement with the levels of Vitamin A in a proximal to distal gradient, which correlates with the abilities of DC to produce RA depending on their location in the intestinal tract (177). For instance, DCs obtained from the proximal small bowel express higher *raldh2* mRNA levels compared to distal small bowel-derived DCs, correlating with their abilities to induce gut-homing receptors on T cells, conversion of Foxp3^+^ T_REG_, and induction of IgA class switch on B cells (177). These examples highlight the concept that the availability of nutrients locally shapes the immune system. Recent efforts are pointing toward the requirements of vitamin A for proper development of the immune system with the consequent relevance for protection against infection and inflammatory disorders. Future studies exploiting conditional knockout of RA receptors in different immune cell subsets will be crucial to better understand the pleiotropic effects of Vitamin A on the immune system.

#### Vitamin D

In addition to Vitamin A, Vitamin D and its active metabolite calcitriol (1,25(OH)_2_D_3_), can influence the immune system beyond its metabolic role in calcium and phosphate homeostasis. The major source of Vitamin D is the photochemical conversion of 7-dehydrocholesterol to cholecalciferol (Vitamin D3) upon skin exposure to UV light. However, some food such as oily fish are enriched in Vitamin D and serve as a relevant source. Calcitriol, the active form of Vitamin D, results from a complex metabolic pathway involving hepatic and renal enzymatic activity (180). The biologic function of Vitamin D is mediated via binding to the hormone nuclear receptor Vitamin D receptor (VDR) heterodimerized with the RXR, which in turn promotes the transcription of Vitamin D responsive genes by binding their promoter region. Besides its role in regulating intestinal, renal and skeletal absorption of calcium and phosphate and in regulating blood pressure, VDR is also expressed by immune cells that in turn are equipped with the enzymatic machinery to produce calcitriol locally (181). The general positive effect of Vitamin D on the adaptive immune system is anti-inflammatory, as observed in studies showing inhibition of B (182) and T cells proliferation (183) and promotion of a shift from the Th1/Th17 lineage toward a T_REG_/Th2 phenotype (184–186). Consistently, calcitriol action on innate immune cells results in the blockade of production of pro-inflammatory cytokines in CD40L-activated monocytes (187) and the promotion of an immature/regulatory phenotype on DCs (188). The immunoregulatory function of Vitamin D, together with the observation of a higher incidence and prevalence of IBD in northern European countries with lower sunlight exposure, prompted the implication of hypovitaminosis D in the pathogenesis of this disease (189). Indeed, low Vitamin D levels were observed in pediatric Crohn’s disease patients (190) and VDR gene polymorphisms were associated with increased susceptibility to IBD (191). In addition, Vitamin D plays a role in modulating cell homing marker expression. DC-dependent production of calcitriol results in the induction of skin-homing receptors (CCR10) and in the inhibition of gut-homing molecules (α4β7 and CCR9) on activated T cells (192). However, whether this redirection of immune cells is implicated in IBD development remains to be addressed. In the murine setting, IL-10^−/−^ mice (an established mouse model for IBD) crossed with VDR^−/−^ mice displayed exacerbation of colitis and fulminating inflammation when compared with VDR-sufficient control mice (193). Similarly, VDR^−/−^ mice were shown to be more susceptible to Dextran Sodium Sulfate (DSS)-induced colitis due to impaired healing and decreased epithelial junctional complexes, suggesting a role for Vitamin D in the maintenance of the intestinal mucosal barrier (194). Interestingly, VDR^−/−^ mice are characterized by increased bacterial load in the intestinal mucosa. This is most likely due to a VDR-dependent transcriptional regulation of the autophagy gene ATG16L1 in Paneth cells, which are important for the production of antimicrobial peptides (195). Although preclinical and clinical observations point toward an involvement of Vitamin D in the pathogenesis of IBD, further studies addressing whether hypovitaminosis D is a cause or a consequence are needed. IBD patients are characterized by inflammation-dependent intestinal malabsorption of diet-derived Vitamin D (180). However, recent prospective studies suggest that newly diagnosed IBD patients show Vitamin D deficiency whereas higher Vitamin D status correlates with a lower risk of developing Crohn’s disease (196).

#### AhR Ligands

Another relevant dietary constituent enriched in the small intestine is represented by aryl hydrocarbon receptor (AhR) ligands. AhR is a widely expressed basic helix-loop-helix transcription factor, belonging to the PAS domain-containing superfamily. Interestingly, its expression on immune cells plays a pivotal role in the development and differentiation of the immune system (197). Ligands of AhR encompass xenobiotics such as environmental pollutants, endogenous ligands such as tryptophan metabolites (e.g., FICZ) and dietary ligands contained in cruciferous vegetables like broccoli and cabbage (198). Remarkably, a recent report showed that AhR might also act as a sensor of PAMPs by binding bacterial pigmented virulence factors (viz. phenazines from *Pseudomonas Aeruginosa* and naphtoquinone phthiocol from *Mycobacterium Tubercolosis*) and enhancing host immune response against pathogens (199). AhR ligands have been linked to the maintenance, but not the embryogenesis, of intraepithelial lymphocytes (e.g., TCRγδ and CD8αα T cells) (200) as well as differentiation and effector function of Foxp3^+^ T_REG_, Tr1, and Th17 cells (201–203). Recent findings highlighted a crucial role for AhR signaling in the innate lymphoid cells compartment. AhR deficiency dampens the postnatal, but not the fetal, development of RORγt^+^Nkp46^+^ ILC3, thereby affecting IL-22 mediated protection against *C. rodentium* infection (204). Interestingly, AhR-dependent IL-22 expression by ILC3 was shown to be crucial to avoid the expansion of SFB and the induction of Th17 cells in the intestine, suggesting a cross-regulatory role of AhR that directly promotes Th17 cell differentiation and indirectly control their expansion by regulating the activity of ILCs (205). The requirement of AhR-mediated signaling during postnatal development was also outlined by two studies demonstrating an AhR-dependent development of CCR6^−^CD4^−^T-bet^+/−^ RORγt^+^ ILCs to generate cryptopatches and isolated lymphoid follicles (206, 207). Furthermore, AhR expression on peritoneal Mφ has been associated with hyporesponsiveness to LPS achieved by NF-κB inhibition (208). Hence, AhR ligands may target Mφ to maintain tolerance against the non-self repertoire enriched in the GI tract. In addition, AhR negatively regulates NLRP3 inflammasome activity (209), further suggesting a role in dampening intestinal inflammation. To better determine the role of AhR on specific intestinal immune cells, additional studies exploiting an AhR ligand deficient diet, conditional ablation of AhR and a discrimination of microbial derived AhR ligands are needed.

#### Salt (NaCl)

It has recently been shown that salt, which is contained in high concentration in the western diet, induces pathogenic Th17 cells in the intestinal lamina propria (210), and hence providing an explanation for the link between the western life style and the high incidence of IBD observed in developed countries (189). Mechanistically, the serine-threonine kinase SGK1 (serum glucocorticoid kinase-1), which is important in controlling Na^+^ transport and salt homeostasis, is induced in T cells activated in Th17 polarizing conditions. SGK1 signaling blocks Foxo1, which is a repressor of IL-23R. Thus, SGK1-deficient cells display normal primary differentiation of Th17 cells, but impaired IL-17 production upon IL-23 restimulation (210). Consistently, WT mice fed with a high salt diet were characterized by an increased frequency of Th17 in the SI-LP when compared to mice lacking SGK1 in the CD4^+^ compartment (210).

### Commensal-derived food metabolites

Some dietary components require special enzymes for their digestion; those that are lacking in our genome are instead provided by our microbiota (211). Mice reared under germ-free conditions display an altered metabolism with accumulation of undigested fibers and of primary bile acid products (212). The influence of commensal bacteria in regulating the availability of diet-derived nutrients and metabolites and their impact on the immune response have recently been reviewed elsewhere in Ref. (213), therefore we will in brief only discuss recently published results.

#### Short-chain fatty acids

Indigestible dietary fibers, which are present in fruits, vegetables, and cereals, represent the prototype of food metabolites that require bacterial enzymatic digestion in order to be biologically available. Fibers are processed by bacterial fermentation into SCFA products (mainly acetate, propionate and butyrate), which are enriched in the proximal colon in agreement with the higher bacterial load (16). Although butyrate has been associated with a pro-tumorigenic role in the etiology of colorectal cancer (214), it has also been shown that administration of butyrate to mice, which are genetically susceptible to small intestinal cancer and kept under a high fat diet, is able to attenuate tumor progression (215). Overall, the effect of a fiber-enriched diet results in the induction of an anti-inflammatory environment and thus provides a protection against inflammatory and allergic disorders. In line with this, *in vitro* butyrate-treated DCs produced lower levels of inflammatory cytokines, such as IL-12 and IFNγ (216). Furthermore, butyrate modulates intestinal macrophage function through histone deacetylase (HDAC) inhibition and downregulation of the LPS-induced inflammatory cascade (217). Moreover, butyrate binding to GPR109A, which is a receptor for niacin in colonic Mφ and DCs, results in the generation of Foxp3^+^ T_REG_ and IL-10 producing Tr1 cells (218). *In vivo*, the most prominent effect of SCFAs is the expansion of Foxp3^+^ T_REG_ cells in the colonic lamina propria. SCFA-mediated effects on the immune system are mostly exerted through inhibition of HDAC and signaling downstream GPR43 and GPR41 receptors (213). A SCFA-enriched diet mediates the expansion of Foxp3^+^ T_REG_ in the colon in a cell intrinsic and GPR43-dependent manner (219), whereas previous results showed the same expansion mediated by inhibition of deacetylation of the Foxp3 promoter (220). Interestingly, from a metabolic point of view, Th17 cells, differently from Foxp3^+^ T_REG_, are dependent on the glycolytic pathway (221). A recent study showed that *de novo* fatty acid synthesis is crucial for Th17 cell differentiation, whereas T_REG_ are mostly induced by fatty acid uptake, thus providing a concomitant metabolic explanation of the phenotype observed *in vivo* upon changes in the dietary intake (222). Consistently with the enlargement of the Foxp3^+^ T_REG_ pool in the colon, mice fed with a fiber enriched diet showed decreased T cell transfer-mediated colitis, which depends on a direct effect of SCFA on Foxp3^+^ T_REG_ rather than *de novo* Foxp3^+^ T_REG_ generation (219). Using a chemically induced murine model for colitis, Berndt and colleagues demonstrated that the route of administration is crucial to determine a protective or a harmful role of butyrate (223). The analysis of feces from human patients with IBD compared with healthy subjects revealed decreased levels of acetic, butyric, and propionic acids, thus suggesting a protective role of SCFA in the pathogenesis of IBD (224). Moreover, the fecal microbiota of patients with IBD showed decreased levels of *F. Prausnitzii*, a bacterial species of Firmicutes involved in dietary fiber digestion (225). Nevertheless, the effect of a fiber enriched diet in the treatment of IBD patients has shown opposite results (226–228), possibly depending on the different experimental protocols used in the different studies and on the selection of the patients cohorts (227). A better understanding of the local versus systemic effect mediated by this metabolite is needed to achieve a deeper insight in the mechanisms of action and the possible consequences in the shaping of the immune response in the GI tract and in other compartments of our body. In line with this, a fiber-rich diet is reflected by an increase in the serum level of SCFAs and is associated with a reduced susceptibility to allergic airway inflammation in a GPR41-dependent manner. This protective effect appeared to be mediated by an increased proliferation of CDPs and MDPs in the BM and the subsequent seeding of the lungs with DCs characterized by an increased phagocytic potential but a decreased ability to produce type 2-promoting cytokines (229). Whether this extra-intestinal effect of dietary metabolites is dependent on a direct sensing of SCFAs by BM precursors or whether it is an indirect effect mediated by education of immune cells in the intestine, followed by delivery of signals to the BM, still needs to be investigated.

#### Oxysterols and Bile Acids

Food enriched in oils and fat is an important source of dietary lipids, mainly triglycerides and cholesterol, which exert important roles in regulating the immune system (230). Metabolism of cholesterol gives rise to oxysterols and bile acids, key players in the regulation of nutrient absorption and lipid digestion through the activation of the nuclear receptors LXR (LXRα and LXRβ) and FXR, respectively. Commensal bacteria are important regulators of the synthesis of bile acids by converting primary into secondary bile acids and by favoring their excretion. Bile acids binding of FXR-RXR heterodimerized receptor expressed on lamina propria CD11b^+^ Mφ results in a decreased induction of pro-inflammatory genes, like IFNγ, IL-6, TNF-α, and IL-1β. Therefore, mice treated with an FXR agonist displayed a milder phenotype of inflammation during DSS-induced colitis (231, 232). Similarly, LXR activation on LPS treated Mφ results in the inhibition of COX-2, NOS, and IL-6 expression with the subsequent reduced inflammation in an *in vivo* model of dermatitis (233). Consistently, LXR-deficient Mφ display an aberrant capacity of apoptotic cell clearance associated with induction of inflammation and breakdown of self-tolerance (234). On the other hand, LXRβ deficiency was associated with an enhanced proliferative capacity of T lymphocytes and lymphoid hyperplasia, implying the importance of LXR and sterol metabolism in lymphocytes homeostasis (235). Furthermore, LXR activation has been associated with impaired differentiation of Th17 cells and protection against experimental autoimmune encephalomyelitis (EAE) in mice (235). Given the need to maintain intestinal homeostasis, metabolism of cholesterol might be crucial to counteract pro-inflammatory exogenous insults highly present in the GI tract. However, the specific target cells sensing oxysterol and/or bile acid to exert homeostatic function is still unresolved. Interestingly, a recent study demonstrated that naturally occurring oxysterols (7β, 27-OHC, and 7α, 27-OHC) are strong RORγt agonists, hence enhancing Th17 differentiation *in vitro* in both human and mice (236). This study was also further substantiated by *in vivo* findings. Mice deficient for CYP27A1, an enzyme responsible for bile acid synthesis and 27-OHC production, showed diminished numbers of Th17 cells among splenic CD4^+^ and γδ T cells (236), suggesting that oxysterols might, independently of LXR, modulate intestinal Th17 homeostasis.

Overall, the concept of diet as an active player involved in shaping our immune system rather than just being a mere source of energy is broadly accepted. The study of nutritional regimens in different areas of the world paired with the differential incidence of inflammatory intestinal disorders is currently providing important insights in their pathogenesis and etiology. Current efforts point to uncover dietary derivatives associated to cellular and molecular changes leading to intestinal inflammation or reestablishing homeostasis. Moreover, a better understanding of diet-mediated immune effects will be crucial to exploit changes in dietary habits as an important complementary therapeutic approach for the treatment of inflammatory disorders affecting the GI tract.

## Oral Tolerance to Food Antigens

As mentioned, the gut mucosa is constantly exposed to commensal microflora and food constituents. Therefore, efficient suppressive mechanisms are needed to avoid undesired inflammatory responses against these innocuous antigens. Oral tolerance is a process characterized by local and systemic hyporesponsiveness to antigens administered via the intestinal mucosa. Defects in proper induction and/or maintenance of oral tolerance might underlie pathological immune responses not only in the gut (e.g., IBD and food allergy), but also beyond the gut (e.g., multiple sclerosis).

### Cellular mechanisms of oral tolerance

**Figure 4:**
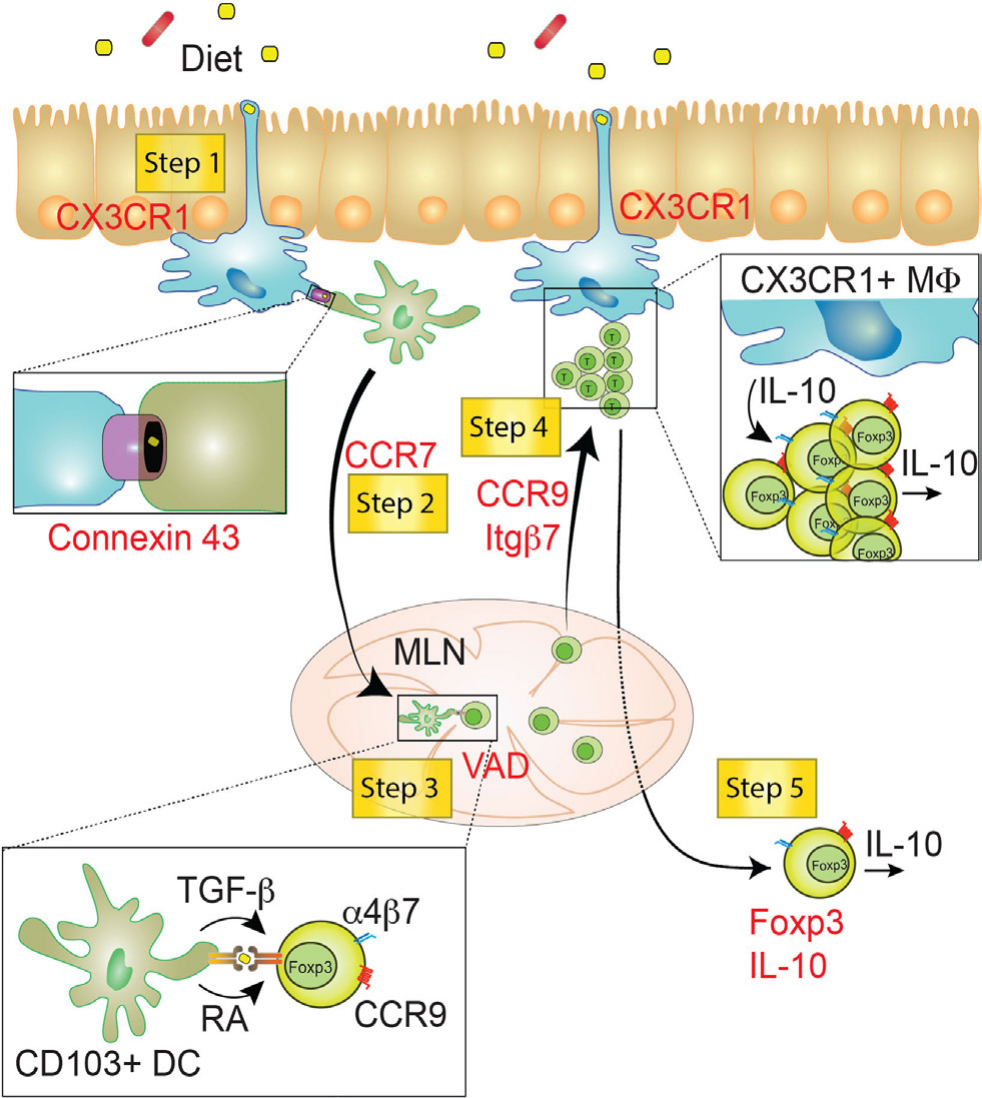
**Step-wise induction of oral tolerance**. Five different steps can be characterized during the induction of oral tolerance (yellow squares). These steps have been identified using key deficiencies (red), such as genes or vitamins. *Step 1*: CX3CR1^+^ macrophages (Myeloid cells depicted in blue) might sample food antigens though dendrites crossing the IEC barrier. Food antigens may then be transferred via Connexin 43^+^ gap junctions from macrophages to migratory CD103^+^ DCs (Myeloid cells depicted in green). *Step 2:* Food antigen-bearing CD103^+^ DCs migrate to the MLN in a CCR7-dependent manner and mediate the priming of Foxp3^+^ T_REG_ through the production of TGF-β and retinoic acid (RA). *Step 3:* newly generated T_REG_ are endowed with the ability to migrate to the small intestine lamina propria. This process relies on the induction of gut-homing receptors (CCR9 and α4β7) in a Vitamin A-dependent manner. *Step 4:* T_REG_ cells migrate to the SI-LP, where they proliferate and acquire tolerogenic properties, likely involving IL-10 produced by CX3CR1^+^ macrophages. *Step 5:* fully tolerogenic T_REG_ generated in the SI-LP may be released in the circulation preventing peripheral inflammation in a Foxp3- and IL-10-dependent manner. VAD, Vitamin A deficient diet; MLN, mesenteric lymph node; SI-LP, small intestine lamina propria; Itgβ7, integrin β7; MΦ, macrophage.

The induction of oral tolerance relies on a stepwise active mechanism exploited by intestinal immune cells belonging to the adaptive and the innate immune system (237, 238). It is broadly accepted that induction of antigen-specific Foxp3^+^ T_REG_ in gut draining lymphoid organs, and subsequent migration of these antigen specific Foxp3^+^ T_REG_ into circulation, are key features to establish oral tolerance. However, in recent studies, a multi-step model for the induction of oral tolerance has been proposed, in which prior to entering into the circulation, Foxp3^+^ T_REG_ migrate to the gut mucosa with the purpose to acquire immunosuppressive abilities (Figure 4) (5). The different steps involved in this process include: (1) antigen sampling by innate immune cells; (2) migration to intestinal secondary lymphoid organs (mostly the mesenteric lymph node) and antigen presentation; (3) generation of Foxp3^+^ T_REG_ with gut tropism; (4) migration of Foxp3^+^ T_REG_ to the intestinal lamina propria where they expand and acquire tolerogenic potential; (5) eventual migration of newly generated Foxp3^+^ T_REG_ into circulation and prevention of inflammation in the periphery (5).

### Antigen sampling

Luminal antigen sampling rely on several mechanisms involving different cell types, including Peyer’s Patches-associated M cells, intestinal epithelial cell-mediated intake, and protrusion of dendrites by CX_3_CR1^+^ Mφ across the epithelial layer (237). However, lamina propria Mφ of the uppermost part of the small intestine are less efficient in antigen presentation and, as mentioned above, do not migrate to draining lymph nodes when compared to CD103^+^ DCs. Since antigen presentation by CD103^+^ DCs appeared to be crucial to induce oral tolerance, sampled antigens need to be transferred from CX_3_CR1^+^ Mφ to CD103^+^ DCs. Interestingly, Rescigno’s group demonstrated that orally delivered antigens, internalized and processed by CX_3_CR1^+^ Mφ, could be transferred via Connexin 43^+^ gap junctions to migratory CD103^+^ DCs in order to allow MHC-II mediated antigen presentation within the MLN. Cytoplasmatic peptides are not loaded to MHC-II molecules, thus a mechanism dependent on trogocytosis and transfer of peptide-MHC-II complexes has been proposed to explain the induction of Foxp3^+^ T_REG_ (239). Furthermore, the gel-forming mucins MUC2 activate a β-catenin-dependent tolerogenic pathway in the intestine by binding a Galectin3–Dectin1–FcgRIIB receptor complex on intestinal CD103^+^ DCs. Therefore, mice deficient for MUC2 are no longer able to establish intestinal and systemic tolerance upon oral intake of antigens, implying a crucial immunoregulatory role for the mucus in addition to a mere barrier function (240).

### Migration to the MLN and Ag presentation

Following antigen uptake, DCs need to migrate to intestinal draining lymphoid structures in order to present the antigen to cognate naïve T cells and induce antigen-specific Foxp3^+^ T_REG_. This has been proven using CCR7^−/−^ mice as well as mesenteric lymphadenectomy, which result in inhibition of oral tolerance induction (241). Unlike the CD103^−^ subset, migratory CD103^+^ DCs are endowed with enhanced ability to induce Foxp3^+^ T_REG_ in a TGF-β and RA-dependent manner (7). Wnt–β-catenin signaling in DCs was shown to increase the expression of regulatory cytokines (*Il10* and *Tgfb1*) and the enzymes responsible for RA production (*Raldh1* and *Raldh2*) (242). CD103^+^ DC can also be distinguished by expressing the integrin αvβ8, which converts latent to active TGF-β, hence, further specializing them to induce Foxp3^+^ T_REG_ (10). Thus, CD103^+^ DC might play a critical role during the establishment of oral tolerance, as suggested by eliminating RA-producing DC precursors expressing α4β7 in the BM, which result in defective Foxp3^+^ T_REG_ induction (179). Interestingly, among BM hematopoietic cells, other precursors are characterized by the expression of α4β7, including ILC progenitors (31). Hence, it is tempting to speculate that RA regulates ILC homing to the intestine and the consequent induction of oral tolerance. Indeed, a role for intestinal ILCs has recently been proposed in the establishment of tolerance toward food antigens. LTi and NKp46^+^ ILC3s production of GM-CSF was shown to be essential for the maintenance of resident DCs and for their ability to synthetize RA and produce TGFβ. Noteworthy, GM-CSF production by RORγt^+^ ILCs was dependent on Mφ sensing microbial signals and subsequent production of IL-1β (243), implying a role for the microbiota in this process. Furthermore, monocolonization with Clostridia confers allergy protection by inducing ILC3-mediated production of IL-22 and by regulating allergens access through the epithelial cell layer (244).

### Generation of T_REG_ with gut tropism

Interestingly, CD103^+^ DC also induces the expression of gut-homing receptors, namely CCR9 and α4β7, conferring small intestine tropism to newly generated Foxp3^+^ T_REG_ (7). In agreement with the role of RA in inducing gut-homing tropism, mice fed with a VAD are impaired in the establishment of oral tolerance. However, the proportion of intestinal Foxp3^+^ T_REG_ found in VAD and control mice is comparable, suggesting that RA plays a pivotal role in the induction of gut tropism on T cells rather than in the generation of Foxp3^+^ T_REG_ (5)_._

### Migration of T_REG_ to SI-LP

After the generation of Foxp3^+^ T_REG_ within the MLN, a previously unpredicted additional step has been recently described: migration to the small intestine lamina propria (SI-LP) rather than to the periphery. The observation that RA induces the expression of α4β7 and CCR9 on Foxp3^+^ T_REG_, prompted two different groups to speculate that newly generated Foxp3^+^ T_REG_ are required to migrate to the small bowel in order to establish oral tolerance. Indeed, mice lacking either CCR9 or integrin β7 showed impaired induction of oral tolerance, which was due to the inability of Foxp3^+^ T_REG_ to migrate to the proximal small bowel (8, 9). Foxp3^+^ T_REG_ strongly proliferate in the intestinal lamina propria and most likely they sense CX_3_CR1^+^ macrophage-derived IL-10, which have been proposed to be key processes required during the establishment of oral tolerance (5, 9). Interestingly, Pabst and colleagues showed that CX_3_CR1^+^ deficiency in resident intestinal Mφ abrogates oral tolerance without affecting antigen uptake and presentation, but rather impairing IL-10 production and Foxp3^+^ T_REG_ proliferation in the lamina propria (9). Thus, migration of activated Foxp3^+^ T_REG_ to the small bowel lamina propria seems to be crucial to fully develop a tolerogenic potential. This is in agreement with a growing body of literature proposing the intestine as a site of education or reprograming even for pathogenic T cells (245). In support of this theory, CCR6-deficient Th17 cells, which are impaired to home to the intestine, possess higher pro-inflammatory properties compared to wild type Th17 (245). Moreover, EAE-induced mice (a mouse model for multiple sclerosis) treated with monoclonal anti-CD3 antibodies are protected due to an increased migration of MOG-specific Th17 cells to the duodenum and reduced Th17 cells in the central nervous system (245). In addition, lung-derived pro-inflammatory cells from mice infected with influenza accumulate in the small intestine where they mediate gastrointestinal symptoms (246). Although the authors did not demonstrate that blocking this migratory pattern could affect the disease in the lungs, it might be possible that influenza-induced gastrointestinal inflammation reflects the cost of reducing inflammation in the lungs and resolving viral infection. Overall, oral tolerance relies on the induction of gut-tropic Foxp3^+^ T_REG_ specific for fed antigens.

Failure to prompt an effective regulatory response is associated with development of food allergy characterized, predominantly, by a Th2 response. For example, mice carrying a mutation in the IL-4Rα chain that enhances the IL-4R signaling pathway are more prone to develop allergic sensitization to oral antigens, which is associated with Foxp3^+^ T_REG_ reprogramming toward a Th2 cell-like phenotype (247). Moreover, polymorphisms in the IL4RA locus have been linked to the pathogenesis of human food allergy, and analogously, reprogramed cells (Foxp3^+^ T_REG_ → Th2) were also found in the peripheral blood of children with food allergy (247).

### Deletion and anergy

A possible alternative step has been proposed for the induction of oral tolerance, involving deletion or anergy induction rather than the generation of regulatory T cells. These different outcomes appear to depend on the different doses of antigens orally administered. In particular, feeding low doses of antigens results in the differentiation of regulatory T cells (as described earlier). Although the most characterized subtype of regulatory T cells is CD4^+^ Foxp3^+^ peripherally induced T_REG_, a role for Tr1 Foxp3^−^ IL-10-producing T_REG_ and Th3 Foxp3^−^ LAP^+^ TGF-β-producing T_REG_ has been described in the low-dose induction of OT. *In vivo*, treatment with anti-LAP mAb was recently shown to abrogate anti-CD3-induced OT in mice (248). Conversely, administration of high doses and thus systemic spreading of the antigen relies on the induction of apoptosis or anergy in effector cells. High-dose feeding with myelin basic protein (MBP) in mice, transgenic for MBP-specific TCR, induces an initial wave of activation of CD4^+^ T cells and TCR downregulation, followed by anergy and consequent deletion (249). The molecular mechanism underlying the induction of anergy relies on an active process that involves the activation of anergy-associated genes. Ca^++^/Calcineurin signaling promotes the activation of NFAT that imposes an anergic program if prevented to bind its transcriptional partner AP-1 (250). Among genes that are upregulated on anergic T cells, GRAIL, an E3 ubiquitin ligase, is crucial for the induction of OT. GRAIL^−/−^ mice, genetically engineered to express a TCR specific for the MHC-II restricted peptide of ovalbumin protein (OT-II) on CD4^+^ T cells, are prevented to establish tolerance to fed OVA antigen (251). Deletion of effector cells is important to avoid reactions against food antigens. Oral gavage of high doses of allergen, in a mouse model of allergic contact dermatitis, promotes plasmacytoid DCs-mediated deletion of Ag-specific CD8^+^ T cells in the liver and in the MLN. Residual effector CD8^+^ T cells, that have escaped this first round of tolerance induction, are then subjected to suppression by activated T_REG_ cells, generated as described earlier, in the mucosal associated lymphoid tissues (252).

Most of the studies reported above, aimed at defining OT mechanisms are conducted exploiting TCR-transgenic mouse models that facilitate the tracking of Ag-specific T cells *in vivo*. A general concern about the use of these models is that they are far from being physiologic, since TCR-transgenic T cells recognize their specific antigen with high avidity and generally outnumber the normal repertoire of TCRs specificities. In particular, this might have an impact on studies focused on the induction of anergy and deletion that are influenced by the strength of TCR engagement and signaling.

## Concluding Remarks

In order to exert all its biological functions, our body demands energetic fuel that is mainly provided by nutrition. The first diet is represented by breast milk, which is subsequently replaced by solid food intake. It is well accepted that orally delivered nutrients exert a profound biological role in shaping our intestinal immune system, with consequences affecting our susceptibility to gastro-intestinal and extra-intestinal disorders. Choosing the diet-regimen in all the stages of our life, starting from breast-feeding (or formula) to the solid diet later on, may have immunological implications, which are not completely understood.

For instance, breast milk-mediated transfer of maternal immune-regulating factors assists the development of the immature immune system of the infant, and help in the protection against the first external pathogenic insults. Numerous breast milk components have been described so far, however, studies aimed at dissecting the molecular and cellular mechanisms involved in the fitness of the immune system, including immune maturation and induction of tolerance, are needed.

In contrast, solid food intake is no longer characterized by the presence of preformed immune mediators, but instead dietary metabolites are endowed with immune-shaping properties that influence the composition of a mature immune system. Several immune effects of food components have been characterized in the last few decades. Even though genetically engineered mice and dietary regimens deprived or supplemented with a specific food metabolite in mouse models have been instrumental, we urgently need to corroborate these findings in humans, to then translate and design novel therapeutic approaches. Observational and prospective studies in humans may also have an impact on our knowledge on the preventive and therapeutic potential of food. Thus, providing mechanistic insights into how the immune system can be shaped by dietary compounds or breast milk components will offer valuable tools to develop therapeutic strategies against inflammatory disorders or food allergies.

## Conflict of Interest Statement

The authors declare that the research was conducted in the absence of any commercial or financial relationships that could be construed as a potential conflict of interest.
